# Mineral separation protocol for accurate and precise rhenium-osmium (Re-Os) geochronology and sulphur isotope composition of individual sulphide species

**DOI:** 10.1016/j.mex.2020.100944

**Published:** 2020-06-03

**Authors:** N.J. Saintilan, D. Selby, J.W. Hughes, D. Schlatter, J. Kolb, A. Boyce

**Affiliations:** a Department of Earth Sciences, University of Durham, Durham DH1 3LE, United Kingdom; b State Key Laboratory of Geological Processes and Mineral Resources, School of Earth Resources, China University of Geosciences, Wuhan, China; c Bluejay Mining Plc, 2nd Floor, 7-9 Swallow Street, London, W1B 4DE, United Kingdom; d Helvetica Exploration Services GmbH, Carl-Spitteler-Strasse 100, 8053 Zürich, Switzerland; e Department of Geochemistry and Economic Geology, Institute of Applied Geosciences, Karlsruhe Institute of Technology, Adenauerring 20b, 76131 Karlsruhe, Germany; f Isotope Geoscience Unit, SUERC, Rankine Avenue, East Kilbride, Glasgow G75 0QF, United Kingdom; g Department of Earth Sciences, Institute of Geochemistry and Petrology, ETH Zürich, Clausiusstraße 25, 8092 Zürich, Switzerland

**Keywords:** Re-Os-S isotope geochemistry, Sulphide, Mineral separates, Frantz isodynamic separator

## Abstract

A temporal framework for mineral deposits is essential when addressing the history of their formation and conceptualizing genetic models of their origin. This knowledge is critical to understand how crust-forming processes are related to metal accumulations at specific time and conditions of Earth evolution. To this end, high-precision absolute geochronology utilising the rhenium-osmium (Re-Os) radiometric system in specific sulphide minerals is becoming a method of choice. Here, we present a procedure to obtain mineral separates of individual sulphide species that may coexist within specific mineralized horizons in ore deposits. This protocol is based on preliminary petrographic and paragenetic investigations of sulphide and gangue minerals using reflected and transmitted light microscopy. Our approach emphasizes the key role of a stepwise use of a Frantz isodynamic separator to produce mineral separates of individual sulphide species that are subsequently processed for Re-Os and sulphur isotope geochemistry.

• Detailed method and its graphical illustration modified from an original procedure introduced by [1], [2].
• Quality control and validation of monophasic mineral separates made by microscopic investigations and qualitative analysis of aliquots embedded in epoxy mounts.
• The present method, which contributed to the successful results presented in the co-publication by Saintilan et al. (2020), demonstrates why other studies reporting Re-Os isotope data for mixtures of sulphide minerals should be considered with caution.

Specifications Table

**Table:** 

Subject Area	Earth and Planetary Sciences
More specific subject area	*Mineralogy and isotope geochemistry*
Method name	*Monophasic sulphide mineral separates*
Name and reference of original method	*–*
Resource availability	*–*

*Method details

## Core information: sulphide petrography and paragenetic relationships

**Fig. 1:**
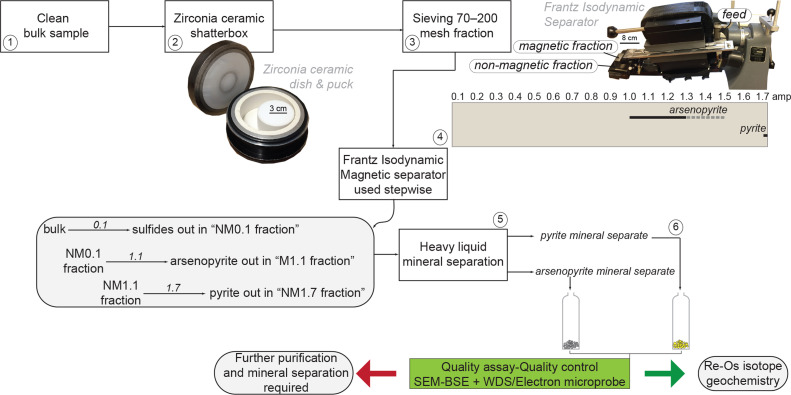
Workflow for the production of pyrite and arsenopyrite monophasic mineral separates from whole-rock samples (modified from [1], [2]). Abbreviations: SEM-BSE: Scanning electron microscopy in backscattered electron mode; WDS: Wavelength-dispersive spectroscopy.

Polished thin sections of the arsenopyrite-pyrite-mineralised samples were studied using transmitted and reflected light microscopy in order to establish the paragenetic relationships and constrain the workflow (Fig. 1) for optimum mineral separation of individual sulphides through modification of the protocol presented by [1], [2]. Paragenetic relationships between sulphide minerals make the basis to determine what currents need to be apply successively on the Frantz Isodynamic Separator to sequentially separate out the individual sulphide species.

## Preparation and quality control of sulphide mineral separates

All sulphide samples were cut into slabs that were thoroughly cleaned using silicon carbide grit, milli-Q water and ethanol to remove any metal traces potentially introduced through hammering or sawing (NB: based on criteria of mineral grain size, sample TTQ-02 was split in two sub-samples to produce two arsenopyrite mineral separates of coarse-grained and very coarse-grained arsenopyrite). These clean sulphide-bearing samples were then crushed using a zirconia ceramic dish and puck and sieved through disposable home-made nylon sieves to produce 70‒200 mesh size fractions (Fig. 1). Each 70‒200 mesh size fraction was washed with milli-Q water using a drip bottle to create a turbulent flow that would wash the particles finer than 74 µm (200 mesh size). Each fraction was then rinsed with ethanol and dried overnight in an oven at 60 °C. In the next step, a Frantz Isodynamic Separator was used to separate magnetic (M) and non-magnetic (NM) sub-fractions by applying successively increasing currents with 15° side slope and 10° forward slope: 1) a 1.1 amp current to collect arsenopyrite (M1.1 fraction), 2) further treatment of the NM1.1 sub-fractions at a 1.7 amp current to collect pyrite in the NM1.7 sub-fraction. The sulphide species were then further purified from any remaining gangue minerals by heavy liquid separation using Sodium Polytungstate (SPT, specific gravity of 2.82 ± 0.02). The sulphide mineral separates, which were collected in filter paper in a funnel, were rinsed with Milli-Q water (previously heated up at ca. 40°C) for a minimum of ten times to remove the heavy liquid prior to a final rinse with ethanol and drying overnight in an oven at 60 °C.

An aliquot of each arsenopyrite and pyrite mineral separate was embedded in epoxy to operate a quality control of these mineral separates (i.e., arsenopyrite-only or pyrite-only). The mounts were studied by reflected light microscopy. We recommend the use of a scanning electron microscope (SEM) operated in backscattered electron mode (SEM-BSE) if doubts persist regarding the quality of mineral separates. Qualitative observations may then be complemented by point wavelength-dispersive spectroscopy (WDS) quality control analyses of arsenopyrite and pyrite in the mounts using the following suite of elements: S, Fe, Ni, Cu, As.

## Declaration of Competing Interest

The authors declare that they have no known competing financial interests or personal relationships that could have appeared to influence the work reported in this paper.
